# Identification and Characterization of Malolactic Bacteria Isolated from the Eastern Foothills of Helan Mountain in China

**DOI:** 10.3390/foods11162455

**Published:** 2022-08-15

**Authors:** Jingxian Sun, Yuzi Ge, Xiaobo Gu, Ruyi Li, Wen Ma, Gang Jin

**Affiliations:** 1 School of Agriculture, Ningxia University, Yinchuan 750021, China; 2 School of Food and Wine, Ningxia University, Yinchuan 750021, China; 3 Engineering Research Center of Grape and Wine, Ministry of Education, Yinchuan 750021, China

**Keywords:** malolactic fermentation, *Lactiplantibacillus plantarum*, enological potential

## Abstract

Malolactic fermentation (MLF) converts malic acid into lactic acid by lactic acid bacteria (LAB). MLF may affect potential wine quality impact as global warming intensifies, and the alcohol in the wine increases, which threatens MLF. *Lactiplantibacillus plantarum* is considered a new generation of MLF starter because of the ability of high ethanol tolerance and good enological characteristics. In this research, 132 LAB strains were isolated from the eastern foothills of Helan Mountain in Ningxia, China. Twenty-one higher ethanol tolerance isolates were obtained by 15% (*v*/*v*) ethanol preliminary screening. They were identified by 16S rRNA sequencing and differentiated by randomly amplified polymorphic DNA (RAPD). Stress factors include ethanol, pH, and SO_2_, and the combination of stresses was used to screen stress-tolerance strains. β-D-glucosidase activity, MLF performance, and biogenic amine content were tested to evaluate the enological characteristics. GC-MS detected the volatile components of the wine after MLF. The results showed that twenty strains were identified as *L. plantarum*, and one strain was *Lentilactobacillus hilgardii*. Especially, the strains of A7, A18, A23, A50, and B28 showed strong resistance to high ethanol, low pH, and high SO_2_. A7, A50, and B28 showed better β-D-glucosidase activity and thus were inoculated into cabernet sauvignon wines whose ethanol content was 14.75% (*v*/*v*) to proceed MLF. A7 finished MLF in 36 d, while the control strains *Oenococcus oeni* 31-DH and *L. plantarum* BV-S2 finished MLF in 24 d and 28 d, respectively. Nevertheless, A50 and B28 did not finish MLF in 36 d. The data showed that A7 brought a more volatile aroma than control. Notably, the esters and terpenes in the wine increased. These results demonstrated the potential applicability of *L. plantarum* A7 as a new MLF starter culture, especially for high-ethanol wines.

## 1. Introduction

Malolactic fermentation (MLF) plays a vital role in winemaking [1,2,3]. It is completed by a kind of malolactic bacteria (MLB) that can metabolize L-malic acid to L-lactic acid. MLF can be started by MLB, which, from the surface of the grape, it is not always successful [4]. This is due to wine’s poor and complex habitat conditions (high ethanol, low pH, high SO_2_). It results in the inhibition or even inactivation of wild LAB, thus affecting the progress of MLF. Based on the highly genetic polymorphisms and mutants of *Oenococcus oeni*, the starter of commercial MLB is mostly *O. oeni* [5]. Usually, *O. oeni* is the dominant flora in wine at the beginning of spontaneous MLF. However, due to the influence of climate change, the ethanol of wines is getting higher and higher. *O. oeni* strains cannot survive well in high-ethanol wines, which make the start and finish of MLF become more and more difficult. More and more research found that *Lactiplantibacillus plantarum* can strongly tolerate high ethanol [6,7,8]. Therefore, isolating and screening some potential *L. plantarum* strains have great significance for the wine industry.

*L.plantarum* is a common MLB in wines. For its good fermentation and safety characteristics, *L. plantarum* is widely used in food fermentation [5,9,10,11]. *L. plantarum* has good resistance to environment stress, such as salt, acid, and ethanol stress [12]. *L. plantarum* may survive in wine environments with up to 17% (*v*/*v*) ethanol, and it has numerous benefits to MLF and wines compared with *O. oeni*, including fewer nutritional needs and faster growth rate and bacteriocin metabolism [6,7,13,14,15]. It helps improve wine color and is an anti-browning agent for wine [16,17]. *L. plantarum* is rich in various enzymes, including esterase, citrate lyase, β-glucosidase, proline aminopeptidase, and phenolic acid decarboxylase (PAD), and due to the enzyme activity, some strains alter the chemical composition of wine by metabolizing synthetic aroma precursors [18]. This brings more possibilities for MLF. Esterases can help synthesize or hydrolyze esters and can contribute to wine aroma [19]. β-Glucosidase promotes glycoside hydrolysis and enriches wine aroma substances. PAD is associated with phenolic acid formation. The presence of β-glucosidase and phenolic acid decarboxylase could allow this species to be used in the future to alter the aroma profile of wine [20]. Tannase can hydrolyze substrates to produce caffeic acid and quinic acid. It also has substances for chemical stabilization [21]. At the same time, numerous research results show that *L. plantarum* has certain antibacterial activities. Some organic acids and proteins produced by metabolism can act as antibacterial molecules to antagonize other harmful microorganisms [22,23,24,25]. Therefore, as a new type of MLF starter, *L. plantarum* has important value for MLF in winemaking.

In this research, high-ethanol-tolerant *L.* *plantarum* strains were isolated and identified. Stress tolerance, β-D glycosidase activity, enological characteristics, and the biogenic amines produce of the isolates were tested to screen potential MLF starters for high-ethanol wines.

## 2. Materials and Methods

### 2.1. Strains, Medium, and Growth Condition

LAB Strains isolated from petit verdot wines during spontaneous MLF were grown in MRS-AJ medium (20% (*v*/*v*) apple juice was added) and incubated at 28 °C for two days in a CO_2_ incubator.

### 2.2. Isolation of MLB from Wine

Next, 100 μL of wine was serially diluted and then plated to solidify. Then 132 colonies were selected and re-streaked for purification. For screening high-ethanol-tolerant strains, 10^7^ CFU/mL of the isolates were inoculated in an MRS-AJ medium containing 15% (*v*/*v*) ethanol. The viable cell counts of twenty-one strains were higher than 10^7^ CFU/mL and will be used for further experiments.

### 2.3. Identification of MLB by 16S rRNA

DNA of isolates was extracted by bacterial DNA kit (General Biotech, Shanghai, China). The amplification of 16S rRNA was followed and adjusted from the experimental design of Jin et al. [26]. The reaction was performed in a final volume of 25 µL using the following amplification mixture: 1 µL DNA template, 2 µL 10× Taq buffer, 0.8 µL of each primer, 1.6 µL dNTPs (2.5 mM), 1.6 µL Mg^2+^ (25 mM), and 0.1 µL DNA polymerase (5 U/µL) (TransGen Biotech, Beijing, China). Samples underwent an initial 2 min denaturation at 94 °C, followed by 30 cycles of 45 s at 94 °C, 2 min at 66 °C, and 2 min at 72 °C, with the final extension of 10 min at 72 °C. The sequencing results were compared with the known sequences in the NCBI database using the nucleotide local alignment BLAST tool (BLAST, http://www.ncbi.nlm.nih.gov/BLAST (accessed on 15 June 2022)).

### 2.4. RAPD Analysis

The identified *L. plantarum* and control strains (*O. oeni* 31-DH and Bactoferm ^®^Vege-Start 2.0 CN Commercial *L. plantarum*, short for BV-S2) were differentiated by randomly amplified polymorphic DNA (RAPD). The primers M13 (5′-GAGGGTGGCGGTTCT-3′) and M14 (5′-GAGGGTGGGGCCGTT-3′) were used to amplify the RAPD. The reaction was performed in a final volume of 25 µL using the following amplification mixture: 5 µL DNA template, 2.5 µL 10× Taq buffer, 1.5 µL primer, 0.5 µL dNTPs (2.5 mM), 3 µL Mg^2+^ (25 mM), and 0.3 µL DNA polymerase (5 U/µL) (TransGen Biotech, Beijing, China). Samples underwent an initial 3 min denaturation at 94 °C, followed by 35 cycles of 1 min at 94 °C, 1 min at 48 °C, and 2 min at 72 °C, with the final extension of 10 min at 72 °C. PCR products were resolved by electrophoresis in 1.0% (*v*/*v*) agarose gels. The bands of isolated strains were analyzed and compared. Data were reported in a binary format with “1” for the presence of a band and “0” for its absence. For clustering, fragments were analyzed with NTSYS 2.10e software using the Dice similarity coefficient based on the presence/absence of the bands and clustered by the unweighted pair group method with arithmetic mean (UPGMA).

### 2.5. Stress Tolerance of L. plantarum

The isolates were inoculated at 10^7^ CFU/mL in MRS-AJ medium separately supplemented with 13% (*v*/*v*), 15% (*v*/*v*), and 17% (*v*/*v*) ethanol. Anaerobic incubation was carried out at 28 °C for two days. Cell viability was studied by plate counting on MRS agar plates.

The isolates were inoculated at 10^7^ CFU/mL in MRS-AJ medium separately supplemented with pH 3.0, 3.2, and 3.4. Anaerobic incubation was carried out at 28 °C for two days. Cell viability was studied by plate counting on MRS agar plates.

The isolates were inoculated about 10^7^ CFU/mL in MRS-AJ medium separately supplemented with 20 (mg/L), 40 (mg/L), and 60 (mg/L) SO_2_. Anaerobic incubation was carried out at 28 °C for two days. Cell viability was studied by plate counting on MRS agar plates.

The isolates were screened by a combination of factors (ethanol × SO_2_ × pH). A total of nine treatments that include ethanol (11%, 13%, 15% (*v*/*v*)), pH (3.2, 3.4, 3.6), and SO_2_ (20 mg/L, 30 mg/L, 40 mg/L) were set to test the comprehensive tolerance of the isolates. The orthogonal experiment is shown in Table 1, following the computing method of Xia et al. [27].

(1) $$\delta_{Xm}=K_{Xm}-\bar{I}$$

*δ_X__m_* represents the difference between average value under the *X* factor and average value of all experimental results. *K_Xm_* stands for the average value of the results that contain the factor *X* with *m* level. $\bar{I}$ stands for the average value of all the factor *X* test results.

(2) $$R=max\delta_{X1},\;\delta_{X2},\;\delta_{X3}-min\left(\delta_{X1},\;\delta_{X2},\;\delta_{X3}\right)$$

*R* stands for the range.

### 2.6. Determination of the β-D-Glucosidase Activity of L. plantarum

The experimental design of this study was followed Chen et al. [28]. The isolates were inoculated at 10^7^ CFU/mL in an MRS-AJ medium. Anaerobic incubation was carried out at 28 °C for two days. Then, 1 mL of bacteria solution at the logarithmic phase was taken and centrifuged at 10,000× rpm for 10 min. The cells were washed twice in 0.85% NaCl solution and suspended in 0.5 mL of 0.85% NaCl solution.

Then 0.5 mL of citric acid phosphate buffer—p-NPG mixture (pH 5.0, p NPG concentration 5 mmol/L)—was added and mixed in the 0.5 mL suspension cells for reaction. Moreover, the reaction was performed at 37 °C for 1 h, and 2 mL 1000 mmol/L Na_2_CO_3_ solution was immediately added to terminate the reaction. The supernatant was centrifuged at 10,000× rpm for 15 min and transferred to another tube. The absorbance value was detected at Abs_400_ by the microplate reader. The standard curve was obtained by measuring Abs_400_ nm of p-NP solution with different concentrations (0–60 μmol/L).

(3) $$\mathrm{Dry}\;\mathrm{mass}\;\mathrm{of}\;\mathrm{thallus}/(\mathrm{mg}/L)=\frac{{\mathrm{Abs}}_{600}\mathrm{nm}}{2.6168}$$

2.6168 is a fixed value.

β-glucosidase activity was defined as the amounts of p-NP-producing substances per gram of thallus (dry mass) per minute/(μmol/(g·min)).

### 2.7. MLF Assay and Basic Chemical Parameters

Cabernet sauvignon wines without MLF were used to perform an MLF assay. After filtering by 0.22 μm filter membrane, 10^7^ CFU/mL bacteria were inoculated in wine. The wine without MLF was used as the control. The samples were collected every four days to determine the changes of L-malic acid content. 

Ethanol, pH, total sugar, hue, and chromaticity of wines before and after MLF were determined by the Chinese national standard test methods (GB/T 15038-2006, the analytical methods of wine and fruit wine). Volatile acid, titratable acid, L-malic acid, and glycerol were measured enzymatically with Analyzer Y15 (Biosystems, Food Quality, Barcelona, Spain).

### 2.8. Biogenic Amine

Biogenic amines were determined by the Chinese national standard test methods (GB/T 5009. 208-2016, the determination of biogenic amine in foods).

### 2.9. Analysis of Volatile Compounds

The volatile compounds of wines were identified using gas chromatography–mass spectrometry (HS-SPME-GC-MS) (Agilent 7890B gas chromatography in tandem with an Agilent 7000D mass spectrometer) (Agilent Technologies, Santa Clara, CA, USA) with an autosampler system (PALRSI 85) (CTC Analytics AG, Zwingen, Switzerland). The separation was performed on a DB-WAX capillary column (30 m × 0.25 mm × 0.25 μm, Agilent, USA). Next, 5 mL of wine, 1.5 g NaCl, and 10 μL 4-methyl-2-pentanol (2.01 mg/L) were taken into 20 mL headspace vial, and the headspace vial was placed in the autosampler. The fiber assembly (23 Ga, 1 cm, 50/30 μm DVB/CAR/PDMS coated) (Supelco, Sigma-Aldrich, Bellefonte, PA, USA) was aged for 10 min at 250 °C. The sample were equilibrated for 5 min at 40 °C and 400 r/min. The fiber assembly was inserted into the headspace bottle to absorb for 30 min at 40 °C and 400 r/min. Then, the fiber assembly was inserted into the GC-MS injection portal and desorbed for 10 min at 240 °C. The injection mode was splitless injection. The GC-MS temperature program was carried out according to Bai et al. [29] as follows: An initial temperature of 45 °C was maintained for 5 min and increased by 3 °C/min to 130 °C/min, followed to 144 °C at a rate of 2 °C/min, then at 5 °C/min speed to 240 °C, and held for 10 min. The MSD transfer line heater was set to 240 °C. The temperature of the ion source was 230 °C, respectively. The mass detector was operated in full scan mode (*m*/*z* 40–300) with electron ionization (EI) mode at 70 eV. Each analysis was performed in duplicate. All compounds were analyzed using the NIST 17 standard mass spectral library. 4-methyl-2-pentanol was used as an internal standard to calculate the relative content of compounds.

### 2.10. Statistical Analysis

All data were expressed as means ± standard deviation. Every sample being analyze three times. Statistical analyses were performed by using the software Origin 2018 (Northampton, MA, USA) and R statistical programming language (Vienna, Austria). The data of volatile compounds were extracted in the Agilent Masshunter Quantitative Analysis software.

## 3. Results

### 3.1. Isolation and Identification of Strains

Overall, 132 LAB strains were isolated from the eastern foothills of Helan Mountain in Ningxia, China. Twenty-one strains were obtained by 15% ethanol screening. Twenty-one strains were preliminarily identified as LAB based on Gram stain positivity, catalase negativity, and cultural and cellular morphology. The 16s rRNA sequences analyzed that twenty strains were *L. plantarum,* and one strain was *Lentilactobacillus hilgardii* (Table 2).

**Figure 1:**
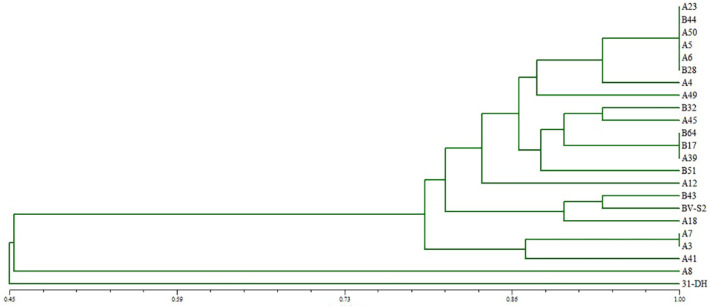
UPGMA dendrogram of twenty-one strains constructed by using RAPD.

UPGMA cluster and dendrogram were obtained from the RAPD analysis of twenty-one isolates and two control strains (Figure 1). All the strains were divided into six different principal clusters. The main clusters included most of the *L. plantarum* strains. The *L. plantarum* strains were clustered in four different principal clusters at a similarity level of 0.76, while *O. oeni* 31-DH and A8 were clustered into a branch, respectively. Overall, the genetic diversity is rich among these *L. plantarum*. *O. oeni* 31-DH and *L.* *hilgardii* A8 reflected the reliability of the experimental method.

### 3.2. Stress Tolerance of L. plantarum

#### 3.2.1. Tolerance of *L. plantarum* to Ethanol

**Figure 2:**
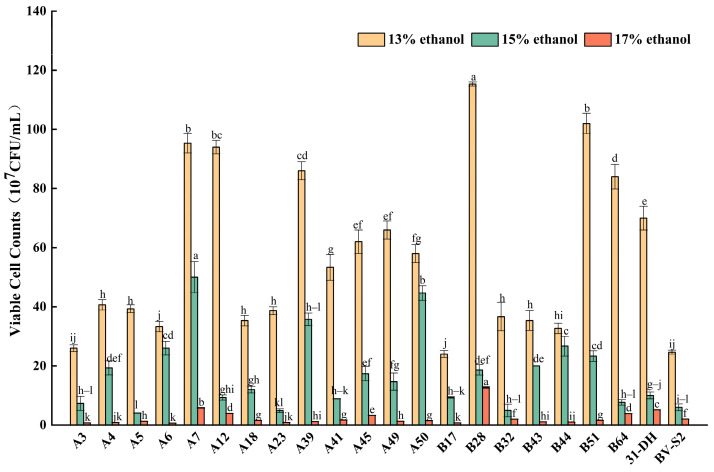
Growth of twenty *L. plantarum* and control strains in MRS-AJ, which contained 13, 15, and 17% (*v*/*v*) ethanol, respectively. Different small letters indicate significant differences according to the LSD Fisher test (*p* < 0.05).

Ethanol can strongly inhibit the growth of MLB and influence MLF. In this research, *L. plantarum* could grow well in MRS-AJ broth with 13% (*v*/*v*) and 15% (*v*/*v*) ethanol. A7, A50, B28, B44, and B51 showed good resistance to ethanol stress. When the ethanol reached 17% (*v*/*v*), the resistance of different strains to ethanol was more different. Most *L. plantarum* could not maintain normal growth after stress. Their tolerance to high ethanol was weak. However, A7 and B28 could still proliferate normally. The viable cell count could reach 1 × 10^8^ CFU/mL, which was different from control strains and other strains (*p* < 0.05). A7 and B28 had strong tolerance to high ethanol stress (Figure 2).

#### 3.2.2. Tolerance of *L. plantarum* to pH

In the process of MLF, pH is an important factor affecting the MLB. pH had a significant effect on the normal growth and metabolism of the strains. Moreover, the growth of the strains decreased with the decrease in pH. There were significant differences in the viable cell counts among different strains at the same pH (*p* < 0.05).

**Figure 3:**
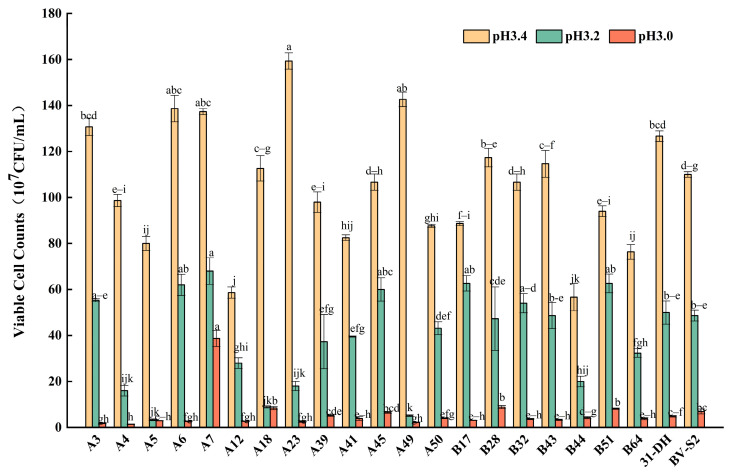
Growth of twenty *L. plantarum* and control strains in MRS-AJ, which contained pH3.4, 3.2, and 3.0, respectively. Different small letters indicate significant differences according to the LSD Fisher test (*p* < 0.05).

When *L. plantarum* were cultured at pH 3.4, they could grow normally, and the cell viable count reached 6 × 10^9^ CFU/mL. When the pH decreased to 3.2, the growths of the strains were inhibited. Among them, the viable cell counts of A3, A6, A7, A45, B43, and B51 were higher than the control strains, showing better tolerance to acidity. When the pH decreased to 3.0, the viable cell counts of most strains decreased by order of magnitude. Nevertheless, it could be maintained at 1 × 10^7^ CFU/mL. They could still maintain their activity under the environment of pH 3.0 except that viable cell counts of A7 and B51 were significantly higher than those of the control strains. The performance of the other strains was similar to or worse than that of the control strains. The overall analysis showed that the strains could grow normally at pH 3.4 and 3.2. A7 and B51 could maintain their growth under the pH of 3.0. They had a better tolerance for acidity (Figure 3).

#### 3.2.3. Tolerance of *L. plantarum* to SO_2_

**Figure 4:**
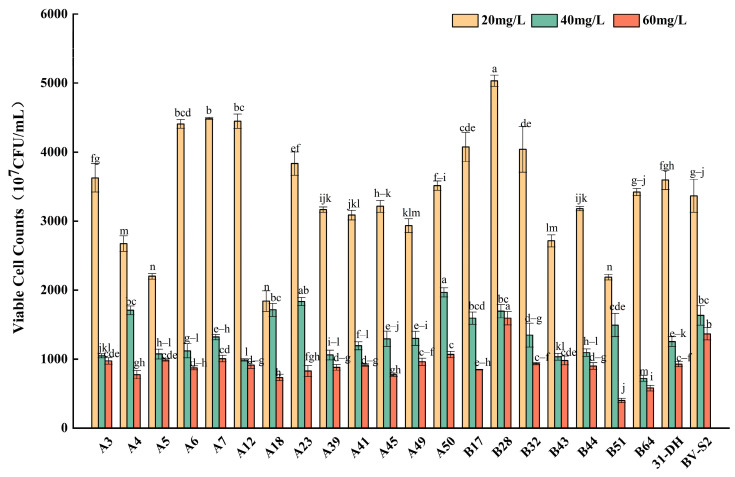
Growth of twenty *L. plantarum* and control strains in MRS-AJ, which contained 20, 40, and 60 mg/L SO_2_, respectively. Different small letters indicate significant differences according to the LSD Fisher test (*p* < 0.05).

Comparing the tolerance of the strains to ethanol, pH, and SO_2_, the growth inhibition of SO_2_ on strains was weaker than that of ethanol and pH. With the 20 mg/L total SO_2_, the viable cell counts of all strains reached 2 × 10^10^ CFU/mL. With the continuous increase of SO_2_, most strains decreased by more than 60%. When SO_2_ increased to 60 mg/L, A7, A50, and B28 showed strong stress tolerance, which was better than or close to the control strains (Figure 4).

#### 3.2.4. Tolerance of *L. plantarum* to Recombinational Factor

**Figure 5:**
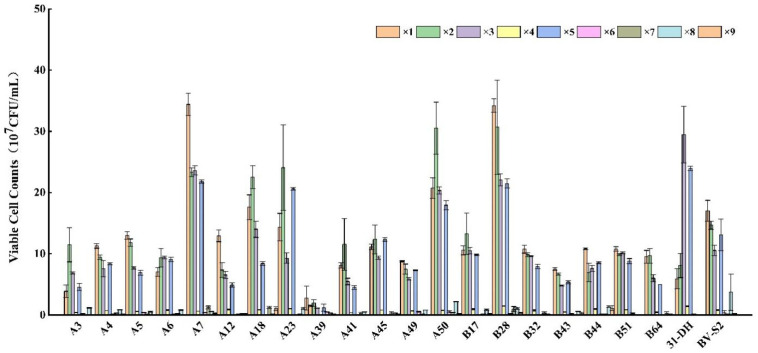
Growth of twenty *L. plantarum* and control strains in MRS-AJ, which contained pH, ethanol, and SO_2_.

MLF is usually carried out in the complex environment of wine, which contains ethanol, organic acids, SO_2_, amino acids, and others. These factors determine the growth and metabolism of LAB in wine. Therefore, investigating the stress tolerance of strains to the compound conditions of ethanol, pH, and SO_2_ is essential for screening good MLB. With the increasing stress intensity of combination of factors, the growth of strains decreased continuously. Furthermore, the growth of A7, A50, B28, and B51 with good resistance to a single factor decreased significantly. The first group of orthogonal treatment had the lowest stress, and the ninth group had the most potent stress. In the ninth group, the viable cell counts of A7, A50, B28, and B51 reached 1.0 × 10^6^ CFU/mL. They showed strong tolerance for the stress (Figure 5).

**Figure 6:**
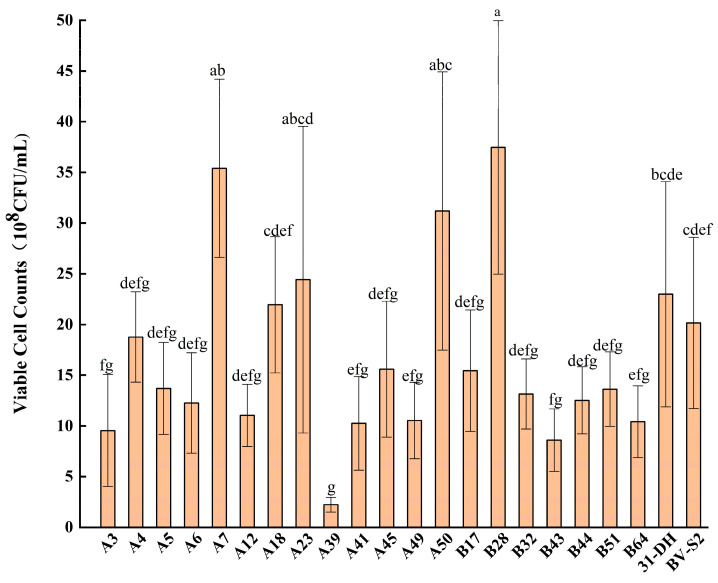
The average value of twenty *L. plantarum* and control strains under nine groups combination of stresses. Different small letters indicate significant differences according to the LSD Fisher test (*p* < 0.05).

The viable cell counts of nine groups were compared by analysis of variance. There was a significant difference in the growth of the isolated strains under combination of stresses (*p* < 0.05) (Figure 6). Among them, the viable cell count of B28 was the highest. The average value was 3.7 × 10^8^ CFU/mL, which was significantly better than the control strains (*p* < 0.05). A7, A18, A23, and A50 were relatively better. The growth of other strains was slightly weaker. The primary and secondary stresses affecting the growth of A7 were ethanol > pH > SO_2_, and the primary and secondary stresses affecting the growth of A18, A23, A50, and B28 were ethanol > SO_2_ > pH (Table 3 and Table 4).

### 3.3. Determination of β-D-Glucosidase Activity

**Figure 7:**
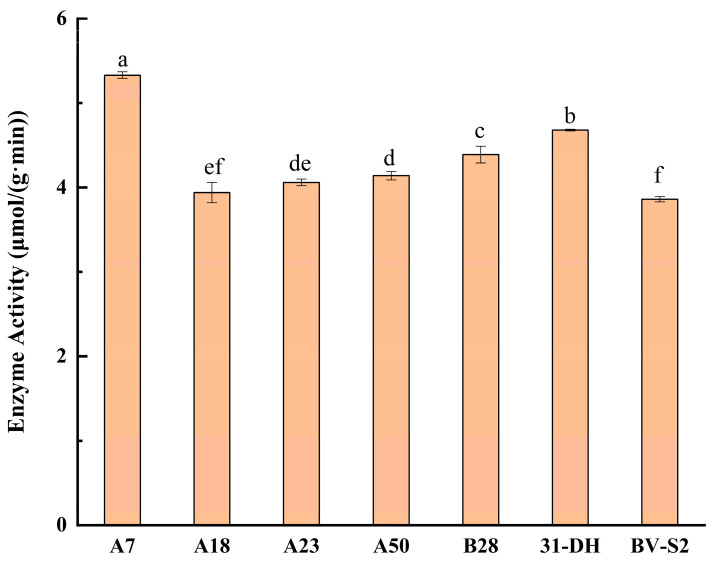
β-D-glucosidase activity of A7, A18, A23, A50, B28, and control strains. Different small letters indicate significant differences according to the LSD Fisher test (*p* < 0.05).

A7, A18, A23, A50, and B28 performed strong stress tolerance and were selected to determine the activity of β-D-glucosidase (Figure 7). The unit of enzyme activity was expressed as μmol/(g·min). All five strains had good β-D-glucosidase activity. A7 had the highest enzyme activity: 5.33 ± 0.05 μmol/(g·min). It was significantly higher than that of the control.

### 3.4. MLF Assay and Basic Chemical Parameters

#### 3.4.1. MLF Assay of Cabernet Sauvignon Wines

**Figure 8:**
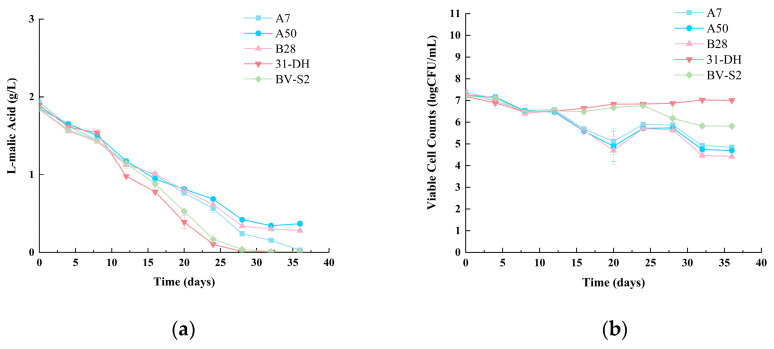
Enological characteristics of *L. plantarum*: (**a**) L-malic acid consumption rate during MLF; (**b**) the viable cell counts during MLF.

Malic acid conversion rate is an important index to evaluate the MLF ability of MLB. L-malic acid was monitored during MLF in cabernet sauvignon wines (Figure 8a). During the MLF process, the L-malic acid concentration fell dramatically with time. Following eight days of inoculation, the L-malic acid concentration in wines fermented by five strains reduced to around 1.4–1.5 g/L. On the 12th day, the L-malic acid concentration in wines fermented with A7, A50, and B28 fell to around 0.6 g/L. Simultaneously, the L-malic acid concentration in wines fermented by *O. oeni* 31-DH and *L. plantarum* BV-S2 fell to around 0.3–0.47 g/L. After 24 days, *O. oeni* 31-DH L-malic acid decreased to less than 0.1 g/L. *O. oeni* 31-DH finished the MLF first. Secondly, on the 28th day, *L. plantarum* BV-S2 finished fermentation. The L-malic acid of A7, A50, and B28 also decreased continuously, and the L-malic acid of A7 decreased to 0.15 g/L on the 32nd day, which showed good fermentation ability. The L-malic acid of A50 and B28 decreased to about 0.4 g/L on the 28th day and remained unchanged, indicating that the strains also had a specific acid-reducing ability.

A7, A50, B28, and the controls were inoculated at about 10^7^ CFU/mL. The cell in the wines during MLF was detected (Figure 8b). After the inoculation, the cell of the strains showed a downward trend. Moreover, the downward trend was the same. Perhaps the wine environment inhibited the growth of strains. When strains adapted to the environment, the number of strains gradually stabilized. On the eighth day, the viable cell counts began to increase. After the 12th day, the viable cell counts of A7, A50, and B28 began to decline gradually. After 20 days of inoculation, the viable cell counts of *O. oeni* 31-DH and *L. plantarum* BV-S2 began to increase. Then the viable cell counts began to decrease from the 28th day. Finally, A7, A50, and B28 remained stable at 10^4^ CFU/mL. *L. plantarum* BV-S2 and *O. oeni* 31-DH were maintained at 10^5^ CFU/mL and 10^6^ CFU/mL, respectively.

#### 3.4.2. Analysis of Basic Chemical Parameters of Wine

Glycerol content did not change significantly before and after MLF. Ethanol contents were between 14.71% (*v*/*v*) and 14.81% (*v*/*v*). After the MLF, the titratable acid of the wines decreased. The lowest titratable acid of *O. oeni* 31-DH was 6.19 g/L. The pH of the wine increased with the decrease of the titratable acid. The pH of A7 (pH = 3.68), A50 (pH = 3.66) and B28 (pH = 3.67) were significantly different from those before MLF. Although volatile acid content increased after MLF, it was lower than the international limit standard. At the same time, MLF affects the color and hue of the wine and can improve its quality. The L-lactic acid in the wine without MLF was 0.08 g/L. After MLF, the L-lactic acid of A7, A50, and B28 was 1.38 g/L, 1.14 g/L, and 1.20 g/L, respectively. Thus, it can be seen that all the strains can metabolize L-malic acid to L-lactic acid. In addition, the total sugar decreased by 0.1–0.5 g/L, respectively, after fermentation. The strains consumed the total sugar in the wine to provide energy for its growth and metabolism (Table 5).

#### 3.4.3. Biogenic Amine Analysis

The formation of biogenic amines is closely related to the metabolism of LAB. Hence, the biogenic amines were evaluated (Table 6). The content is lower than the international limit standard. There was no significant difference in tryptamine and β-phenethylamine tyramine between A7, A50, B28, and control strains after MLF (*p* < 0.05). The productions of A7 of tryptamine, cadaverine, tyramine, and histamine were higher than that in the control strains, and the other two strains were lower than that in the control strains. This may be because the cell counts were always lower than the control strains after inoculation, and amino acid decarboxylase activity was strongly inhibited. Alternatively, the strains lack amino acid decarboxylase-related genes.

#### 3.4.4. Aroma Components Analysis before and after MLF

A total of 27 volatile aroma compounds were detected in non-MLF control wine, and the total amount of volatile aroma compounds was 200 mg/L (Table S1). After MLF, 47 different volatile aroma compounds were detected in different fermented wines, including 29 esters, 8 alcohols, 4 organic acids, 1 terpene, 5 aldehydes, and ketones. The types and contents of volatile aroma compounds in wines increased significantly after MLF.

**Figure 9:**
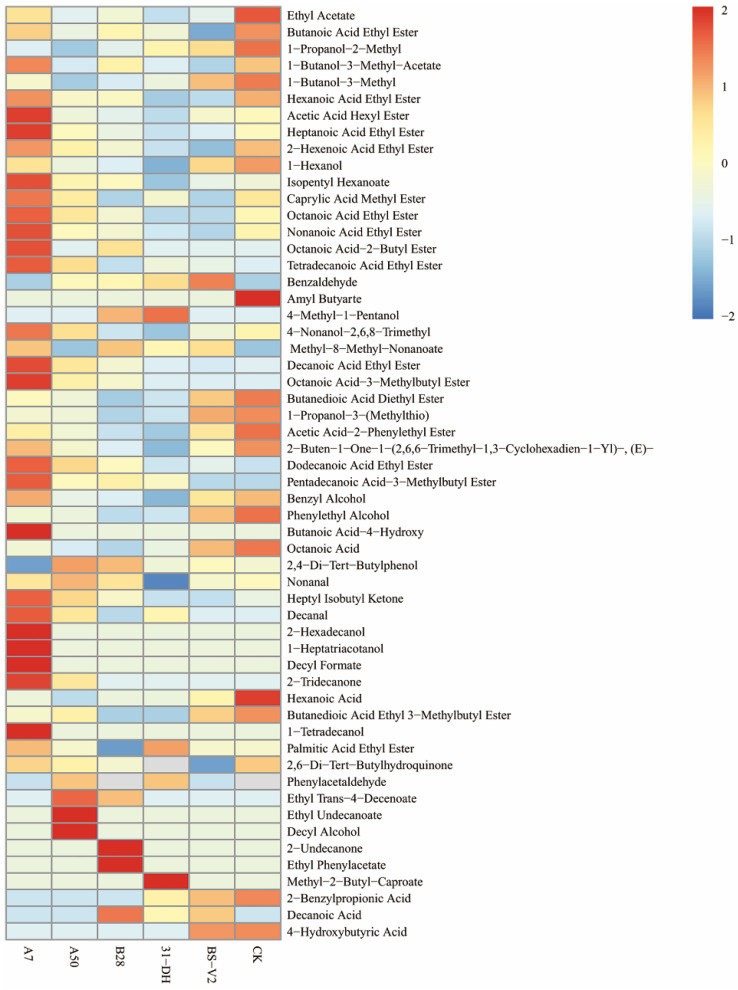
Cluster diagram of volatile compounds for cabernet sauvignon wines.

Compared with *O. oeni* 31-DH, A7, A50, and B28 added nine, eight, and seven kinds of volatile aroma compounds, respectively. Compared with *L. plantarum* BV-S2, A7, A50, and B28 added ten, nine, and seven kinds of volatile aroma compounds, respectively. These volatile aroma compounds were mainly esters. A7 had the most volatile aroma compounds, and the total amount could reach 314.07 mg/L. The volatile aroma compounds of A50 and B28 can reach 244.39 mg/L and 244.12 mg/L (Table S1). A7 increased the content of esters and terpenes. These volatile aroma compounds together constitute the complex aroma of wine (Figure 9).

## 4. Discussion

Twenty strains of *L. plantarum* isolated from petit verdot wines were analyzed by RAPD. It was found that the genomic DNA of the isolated strains was polymorphic. The *L. plantarum* was divided into four clusters, and each category had a different number of branches. The diversity of *L. plantarum* in the same producing area brings more possibilities for MLF. After combination of factors stress on twenty strains of *L. plantarum*, it was found that there were significant differences in stress tolerance among *L. plantarum* isolated from the same producing area. Among them, A7, A18, A23, A50, and B28 grew better and could survive under low acid and high ethanol. Some studies have suggested that the growth limit of *L. plantarum* is pH 3.5, and the ethanol is 8% (*v*/*v*) [30,31]. However, some studies hold other views, saying that *L. plantarum* cannot only survive in the 13% ethanol but also at pH between 3.2 and 3.5 [8,11]. However, it was found that the growth of some *L. plantarum* isolated from the Yinchuan region could be maintained at 10^6^ CFU/mL under pH 3.4 and 15% ethanol, which was different from the above results. However, it can be proved that *L. plantarum* has good ethanol tolerance. The strains showed strong tolerance to high ethanol and good tolerance to pH, SO_2_, and combination of stresses.

Volatile compounds perceptible to the human senses are not detectable in the glycosides that form in wine. Therefore, glycosidase has a major influence on the sensory characteristics of wine [32]. *L. plantarum* is considered to be a new generation of new starter because of its richer enzymatic properties and is gradually used in MLF [33]. Brizuela et al. [2] and Lerm et al. [19] used PCR to amplify genes to prove that β-glucosidase exists in most of *L. plantarum*. Although the existence of this gene does not guarantee the expression in the process of MLF, it can be used as a standard for screening strains and a basis for aroma changes. The environment in wine is complex, while ethanol, acid, and temperature will all affect the enzyme activity [13]. Spano et al. [34] analyzed gene expression under several abiotic stresses, indicating that enzyme activity was significantly regulated by stress. At the same time, the enzyme activity of β-glucosidase is strain-dependent. Not all strains contain this enzyme, and there are differences in enzyme activity [35]. Therefore, screening strains with higher enzymatic activity for MLF has an important impact on aroma characteristics.

All the strains could start MLF typically after being inoculated into the wine. Compared with the wine without MLF, it showed that the MLF of each strain played a role in reducing acid. According to the degradation of malic acid and the change of viable bacteria, it was found that the time of *O. oeni* 31-DH and *L. plantarum* BV-S2 were shorter, and the viable cell counts were higher during MLF. Although the fermentation rate of A7 is slow, it can be finished eventually. However, the MLF could not be finished for A50 and B28. Bravo-Ferrada et al. [36] inoculated *L. plantarum* preadapted in MLO medium into the simulated wine containing 4.5 g/L malic acid. Malic acid consumption increased by 21%~43%. Malolactic enzyme (*mle*) is a key enzyme in MLF process. Miller et al. [37] proposed that *mle* expression was also affected by malic acid content, pH, and ethanol. Different strains show different enzyme profiles. In different environments, the expression levels of *mle* for each strain are also different. Therefore, the rate of acid reduction is affected by many factors. Preconditioning culture and enzyme activity are the key factors affecting malic acid consumption.

In part, the time required for MLF is determined by the recovery time of the viable cell counts. Diez-Ozaeta et al. [38] found that all strains showed a significant decrease in activity or death in the first few days after starting MLF. Nevertheless, it will return to the original count after the first week. In this study, the viable cell counts of all strains dropped throughout eight days. After 12 days, the viable cell counts of A7, A50, and B28 declined to vary degrees. *O. oeni* 31-DH and *L. plantarum* BV-S2 are relatively stable. A7, A50, and B28 decrease and then increase, but MLF can be continued. Genetic differences can explain the differences in acid reduction among strains isolated from the same region. Being able to survive in 17% ethanol does not necessarily help finish MLF. When changing from the nutrient-rich environment to the nutrient-poor environment, and with too many factors affecting growth, the strains will face many difficulties that threaten their growth.

The inhibitory effects of polyphenols in wine on the growth of LAB and the existence of yeast secondary metabolites lead to the incompatibility between yeast and LAB [2]. In comparison, A7 has the potential to become an excellent MLB. López et al. [39] isolated 204 strains from red wine in the Rioja region, of which 98.5% were *O. oeni*. Ruiz et al. [40,41] isolated strains from wines of different years and fermentation stages in different wineries in Spain. Moreover, it identified that *O. oeni* was the dominant strain. The proportion of other strains was between 2% and 10%. Beneduce et al. [42] isolated and identified 150 strains from wines from the Apulia region, most of which were *L. plantarum*. Due to grape variety, wine pH, ethanol, and other factors, the selection of MLB strains becomes more diverse. *O. oeni* is no longer the single choice in the process of MLF. The tolerance of *L. plantarum* to ethanol and its more enzymatic properties make it dominant in the future enology process.

Compared with the aroma composition and content of non-MLF wine, it was found that the composition and content of esters in all wines increased after MLF. Compared with the control strains, the number and content of esters produced by A7, A50, and B28 were relatively higher. Especially A7 had high content in ethyl acetate, isoamyl acetate, ethyl caproate, hexyl acetate, ethyl heptanoate, ethyl trans-2-hexenoate, isoamyl caproate, methyl octanoate, ethyl octanoate, ethyl nonanoate, and isobutyl octanoate. Giving the wine some pleasant aroma, such as fruit, flower, and honey, is essential. Although the total amount of wine alcohols decreased to 78.17 mg/L, 71.31 mg/L, and 73.70 mg/L (Table S1) after MLF, the difference was insignificant compared to control strains. The differences were mainly concentrated in less harmful aromas such as isoamyl alcohol (almond taste, astringent taste) and 3-methylthiopropanol (garlic flavor, raw potato flavor).

Moreover, increase the variety of flower aroma composition. Together with esters and other volatile aroma substances, it enriches wine and reduces undesirable aroma. Compared with control strains, A7 contains benzaldehyde, nonanal, phenylacetaldehyde, 2,4-di-tert-butylphenol and other substances closely related to wine fermentation metabolism was the highest. These substances contribute to the balance of wine taste and the improvement of sensory quality. The variation in aroma content and variety is partly due to β-D glucosidase. Some studies have found that *L. plantarum* is one of the primary sources of β-D glucosidase in oenology [32]. An essential role of β-glucosidase in oenology process is to promote the production of aromatic compounds in wine, thus affecting the aroma and taste of wine [43].

Furthermore, β-glucosidase also changes the composition of phenols in wine by hydrolyzing compounds [44]. A7, A50, and B28 had specific β-D glycosidase activity. It could be seen from the phylogenetic clustering tree that they were all in different branches, which made it possible for MLF to produce more diverse aroma components. In addition to β-glucosidase, it also includes esterase, phenolic acid decarboxylase, citrate lyase, and other enzymes that may affect the flavor of wine and participate in the color improvement of wine [10,11,12,13,14,32,45].

## 5. Conclusions

In this research, 132 strains were identified by molecular identification and tolerance analysis. Three potential strains were selected for enological characteristics analysis. The results showed that the L-malic acid of strain A7 decreased to about 0.1 g/L after 36 days of start-up fermentation. Compared with the control strains, it showed good MLF ability. Compared with the control strains, the variety and content of volatile aroma compounds of strain A7 were richer. Especially the contents of esters and terpenes in wines were significantly increased, which endowed the wines with unique aroma characteristics such as fruit and floral aroma. Therefore, A7 could perform well in wine habitat and potentially be the new MLF starter.

## Figures and Tables

**Table 1:** The orthogonal experimental design.

Levels	Stress		
	Ethanol (%(*v*/*v*))	pH	SO_2_ (mg/L)
1	11	3.2	20
2	13	3.4	30
3	15	3.6	40

**Table 2:** Identification of twenty-one isolated strains by 16S rRNA sequencing.

The Isolated Strains	Identification	16S rRNA Sequencing Similarity (%)
A3, A4, A6, A7, A12, A18, A23, A39, A41, A45, A49, A50, A52, B17, B28, B32, B43, B44, B51, B64	*Lactiplantibacillus plantarum*	99.93–100
A8	*Lentilactobacillus hilgardii*	99.93

**Table 3:** The viable cell counts of orthogonal experiment.

Experimental Number	Ethanol (% (*v*/*v*))	pH	SO_2_ (mg/L)	The Viable Cell Counts (10^7^ CFU/mL)				
				A7	A18	A23	A50	B28
1	1	1	1	24.07	17.60	14.33	16.00	28.53
2	1	2	2	13.33	22.53	24.07	19.13	24.00
3	1	3	3	13.60	14.00	9.27	9.13	15.73
4	2	2	1	0.51	0.82	0.99	0.68	0.86
5	2	3	2	11.47	8.40	20.60	7.60	15.47
6	2	1	3	0.23	0.08	0.09	0.40	0.17
7	3	3	1	0.93	1.22	0.11	0.11	0.69
8	3	1	2	0.49	0.14	1.05	1.18	0.61
9	3	2	3	0.19	1.04	2.77	0.03	0.26

**Table 4:** The orthogonal experiment results.

Strains	Stresses	Levels			R
		1	2	3	
A7	Ethanol	δ_A1_ = 9.80	δ_A2_ = −3.13	δ_A3_ = −6.67	16.47
	pH	δ_B1_ = 1.06	δ_B2_ = −2.52	δ_B3_ = 1.47	3.99
	SO_2_	δ_C1_ = 1.30	δ_C2_ = 1.23	δ_C3_ = −2.53	3.83
A18	Ethanol	δ_A1_ = 10.73	δ_A2_ = −4.21	δ_A3_ = −6.51	17.24
	pH	δ_B1_ = −1.37	δ_B2_ = 0.82	δ_B3_ = 0.56	2.19
	SO_2_	δ_C1_ = −0.76	δ_C2_ = 3.06	δ_C3_ = −2.27	5.33
A23	Ethanol	δ_A__1_ = 7.75	δ_A2_ = −0.91	δ_A3_ = −6.83	14.58
	pH	δ_B1_ = −2.98	δ_B2_ = 1.14	δ_B3_ = 1.85	4.83
	SO_2_	δ_C1_ = −3.00	δ_C2_ = 7.10	δ_C3_ = −4.10	11.20
A50	Ethanol	δ_A1_ = 8.72	δ_A2_ = −3.14	δ_A3_ = −5.59	14.31
	pH	δ_B1_ = −0.17	δ_B2_ = 0.58	δ_B3_ = −0.42	1.00
	SO_2_	δ_C1_ = −0.43	δ_C2_ = 3.27	δ_C3_ = −2.84	6.11
B28	Ethanol	δ_A1_ = 13.16	δ_A2_ = −4.09	δ_A3_ = −9.07	22.23
	pH	δ_B1_ = 0.18	δ_B2_ = −1.22	δ_B3_ = 1.04	2.26
	SO_2_	δ_C1_ = 0.44	δ_C2_ = 3.77	δ_C3_ = −4.20	7.97

**Table 5:** Basic chemical parameters of cabernet sauvignon wines.

Strains	Ethanol (%)	pH	Total Sugar (g/L)	Titratable Acid (g/L)	Volatile Acid (g/L)	L-Lactic Acid (g/L)	Chromaticity	Hue	Glycerol (g/L)
Before MLF	14.75 ± 0.03 ^abc^	3.52 ± 0.01 ^d^	3.63 ± 0.05 ^a^	8.19 ± 0.25 ^a^	0.28 ± 0.08 ^b^	0.08 ± 0.01 ^f^	17.89 ± 2.07 ^a^	0.81 ± 0.54 ^a^	1.03 ± 0.03 ^a^
A7	14.81 ± 0.01 ^c^	3.68 ± 0.01 ^a^	3.53 ± 0.18 ^ab^	7.13 ± 0.15 ^ab^	0.58 ± 0.05 ^a^	1.38 ± 0.01 ^b^	20.50 ± 0.87 ^a^	0.68 ± 0.68 ^bc^	1.10 ± 0.05 ^a^
A50	14.76 ± 0.01 ^b^	3.66 ± 0.01 ^ab^	3.45 ± 0.03 ^ab^	7.13 ± 0.01 ^ab^	0.59 ± 0.11 ^a^	1.14 ± 0.02 ^e^	20.92 ± 2.25 ^a^	0.67 ± 0.02 ^bc^	1.31 ± 0.02 ^a^
B28	14.72 ± 0.01 ^bc^	3.67 ± 0.01 ^ab^	3.22 ± 0.07 ^ab^	7.31 ± 0.05 ^ab^	0.54 ± 0.03 ^a^	1.20 ± 0.01 ^d^	22.14 ± 2.14 ^a^	0.69 ± 0.03 ^b^	1.06 ± 0.24 ^a^
31-DH	14.81 ± 0.01 ^a^	3.65 ± 0.01 ^bc^	3.25 ± 0.29 ^ab^	6.19 ± 0.05 ^b^	0.36 ± 0.01 ^b^	1.45 ± 0.01 ^a^	21.09 ± 1.83 ^a^	0.65 ± 0.07 ^bc^	1.21 ± 0.03 ^a^
BV-S2	14.71 ± 0.00 ^c^	3.62 ± 0.01 ^c^	3.13 ± 0.05 ^b^	7.31 ± 0.01 ^ab^	0.34 ± 0.02 ^b^	1.34 ± 0.02 ^c^	21.62 ± 0.81 ^a^	0.59 ± 0.02 ^c^	1.08 ± 0.01 ^a^

Note: ^a^, ^b^, ^c^, ^d^, ^e^ and ^f^ indicate significance analysis.

**Table 6:** Biogenic amine in cabernet sauvignon wines.

Strains	Tryptamine (mg/L)	β-Phenylethylamine (mg/L)	1,4-Butanediamine (mg/L)	Cadaverine (mg/L)	Tyramine (mg/L)	Histamine (mg/L)
Before MLF	0.64 ± 0.01 ^ab^	1.04 ± 0.08 ^a^	1.46 ± 0.22 ^a^	0.36 ± 0.02 ^a^	0.30 ± 0.02 ^ab^	0.35 ± 0.02 ^a^
A7	0.58 ± 0.04 ^ab^	0.46 ± 0.00 ^bcd^	0.12 ± 0.00 ^c^	1.11 ± 0.09 ^a^	0.58 ± 0.08 ^a^	0.79 ± 0.055 ^c^
A50	0.42 ± 0.01 ^b^	0.18 ± 0.18 ^d^	0.76 ± 0.05 ^b^	0.39 ± 0.17 ^b^	0.14 ± 0.02 ^b^	0.68 ± 0.004 ^b^
B28	0.48 ± 0.04 ^ab^	0.34 ± 0.07 ^cd^	0.86 ± 0.04 ^b^	0.49 ± 0.08 ^ab^	0.11 ± 0.01 ^b^	0.38 ± 0.053 ^b^
31-DH	0.72 ± 0.18 ^a^	0.61 ± 0.04 ^abc^	1.22 ± 0.28 ^ab^	0.62 ± 0.14 ^ab^	0.11 ± 0.00 ^b^	0.38 ± 0.08 ^ab^
BV-S2	0.56 ± 0.02 ^ab^	0.86 ± 0.10 ^ab^	1.26 ± 0.02 ^ab^	0.31 ± 0.00 ^b^	0.11 ± 0.00 ^b^	0.27 ± 0.00 ^ab^

Note: ^a^, ^b^, ^c^, and ^d^ indicate significance analysis.

## Data Availability

Data are contained within the article or supplementary material.

## Supplementary Materials

The following supporting information can be downloaded at: https://www.mdpi.com/article/10.3390/foods11162455/s1, Table S1: Types and contents of volatile compounds in wine samples.
