# Supplementary material for: Identification and Characterization of Malolactic Bacteria Isolated from the Eastern Foothills of Helan Mountain in China
Source: Foods. 2022 Aug 15;11(16):2455. doi: 10.3390/foods11162455 (PMC9407436; doi:10.3390/foods11162455)
Supplement: Supplementary file 1 [file foods-11-02455-s001.zip › foods-1803588-supplementary.pdf]

Table S1. Types and contents of volatile compounds in wine samples.

volatile compounds	content（mg/L）					
	A7	A50	B28	31-DH	BS-V2	Before MLF
Ethyl Acetate	4.70±0.19^{ab}	3.91±0.44^{b}	4.13±0.08^{ab}	3.68±0.07^{b}	3.92±0.14^{b}	5.48±0.23^{a}
Butanoic Acid Ethyl Ester	0.42±0.01^{ab}	0.34±0.01^{ab}	0.38±0.03^{ab}	0.35±0.01^{ab}	0.27±0.09^{b}	0.45±0.01^{a}
1-Propanol-2-Methyl	1.35±0.13^{bc}	1.25±0.05^{c}	1.37±0.12^{bc}	1.50±0.01^{abc}	1.58±0.08^{ab}	1.73±0.07^{a}
1-Butanol-3-Methyl-Acetate	8.14±0.64^{a}	6.30±0.01^{ab}	7.19±0.45^{ab}	6.35±0.25^{ab}	6.00±0.39^{b}	7.73±0.26^{ab}
1-Butanol-3-Methyl	75.33±4.28^{ab}	69.04±3.57^{c}	71.70±2.52^{bc}	73.96±1.71^{bc}	82.17±2.08^{ab}	85.46±0.73^{a}
Hexanoic Acid Ethyl Ester	13.50±0.33^{a}	10.79±1.28^{ab}	10.88±0.49^{ab}	8.70±0.55^{b}	9.15±0.55^{ab}	13.02±0.19^{ab}
Acetic Acid Hexyl Ester	0.27±0.03^{a}	0.16±0.03^{ab}	0.15±005^{ab}	0.13±0.02^{b}	0.17±0.06^{ab}	0.18±0.01^{ab}
Heptanoic Acid Ethyl Ester	0.20±0.05^{a}	0.12±0.02^{ab}	0.10±0.01^{b}	0.08±0.01^{b}	0.09±0.02^{b}	0.12±0.01^{ab}
2-Hexenoic Acid Ethyl Ester	0.37±0.01^{a}	0.31±0.03^{ab}	0.28±0.02^{ab}	0.24±0.02^{ab}	0.21±002^{b}	0.35±0.01^{a}
1-Hexanol	2.75±0.08^{ab}	2.55±0.23^{ab}	2.49±0.01^{ab}	2.34±0.10^{b}	2.77±0.04^{ab}	2.87±0.05^{a}
Isopentyl Hexanoate	0.70±0.64^{a}	0.34±0.01^{ab}	0.28±0.45^{b}	ND	0.18±0.40^{b}	0.22±0.26^{b}
Caprylic Acid Methyl Ester	0.47±0.08^{a}	0.27±0.23^{ab}	ND	0.17±0.10^{ab}	ND	0.29±0.01^{ab}
Octanoic Acid Ethyl Ester	113.13±21.81^{a}	85.81±16.72^{ab}	69.49±7.57^{ab}	51.36±4.21^{b}	51.84±6.41^{b}	77.98±0.58^{ab}
Nonanoic Acid Ethyl Ester	0.68±0.07^{a}	0.38±0.05^{ab}	0.34±0.06^{b}	0.24±0.02^{b}	0.20±0.02^{b}	0.41±0.03^{ab}
Octanoic Acid-2-Butyl Ester	0.14±0.04^{a}	ND	0.07±0.01^{ab}	ND	ND	ND
Tetradecanoic Acid Ethyl Ester	0.61±0.10^{a}	0.46±0.18^{ab}	0.22±0.05^{b}	0.31±0.10^{ab}	0.29±0.02^{b}	0.26±0.02^{b}
Benzaldehyde	ND	0.15±0.03^{ab}	0.16±0.01^{ab}	0.23±0.01^{ab}	0.32±0.02^{a}	ND
Amyl Butyarte	ND	ND	ND	ND	ND	0.02±0.01^{a}
4-Methyl-1-Pentanol	ND	ND	0.03±0.02^{a}	0.04±0.02^{a}	ND	ND
4-Nonanol-2,6,8-Trimethyl	0.52±0.05^{a}	0.44±0.08^{ab}	0.30±0.02^{b}	0.26±0.01^{b}	0.35±0.04^{ab}	0.40±0.02^{ab}
Methyl-8-Methyl-Nonanoate	0.09±0.04^{a}	ND	0.09±0.01^{a}	0.06±0.01^{a}	0.08±0.01^{a}	ND
Decanoic Acid Ethyl Ester	77.08±16.03^{a}	51.98±10.64^{ab}	38.58±4.4.87^{b}	29.57±1.68^{b}	27.70±3.51^{b}	30.12±0.03^{b}

Octanoic Acid-3-Methylbutyl Ester	1.49±0.42^{a}	0.83±0.14^{ab}	0.65±0.11^{b}	0.43±0.03^{b}	0.43±0.05^{b}	0.44±0.08^{b}
Butanedioic Acid Diethyl Ester	0.81±0.04^{abc}	0.74±0.08^{abc}	0.56±0.02^{c}	0.63±0.07^{bc}	0.98±0.01^{ab}	1.10±0.14^{a}
1-Propanol-3-(Methylthio)	0.50±0.02^{ab}	0.48±0.03^{ab}	0.30±0.01^{b}	0.36±0.08^{b}	0.79±0.13^{a}	0.85±0.01^{a}
Acetic Acid-2-Phenylethyl Ester	2.00±0.22^{ab}	1.74±0.16^{ab}	1.49±0.08^{ab}	1.34±0.05^{b}	2.06±0.21^{ab}	2.51±0.17^{a}
2-Buten-1-One-1-(2,6,6-Trimethyl-1,3-Cyclohexadien-1-Yl)-, (E)-	0.28±0.01^{a}	0.21±0.03^{ab}	0.18±0.01^{ab}	0.14±0.01^{b}	0.22±0.03^{ab}	0.30±0.01^{a}
Dodecanoic Acid Ethyl Ester	5.50±2.06^{a}	4.02±1.03^{ab}	2.83±0.37^{ab}	1.54±0.01^{b}	2.00±0.26^{b}	1.48±0.01^{b}
Pentadecanoic Acid-3-Methylbutyl Ester	0.62±0.34^{a}	0.23±0.07^{ab}	0.30±0.02^{ab}	0.21±0.02^{ab}	ND	ND
Benzyl Alcohol	0.56±0.07^{a}	0.40±0.03^{ab}	0.38±0.01^{ab}	0.30±0.01^{b}	0.50±0.02^{ab}	0.55±0.05^{a}
Phenylethyl Alcohol	123.69±14.85^{bc}	119.30±9.20^{bc}	93.52±7.52^{c}	98.59±3.40^{c}	176.75±9.20^{ab}	203.78±19.50^{a}
Butanoic Acid-4-Hydroxy	0.44±0.15^{a}	ND	ND	ND	ND	ND
Octanoic Acid	2.28±0.60^{abc}	1.91±0.25^{bc}	1.62±0.70^{c}	2.13±0.09^{bc}	3.30±0.06^{ab}	3.72±0.35^{a}
2,4-Di-Tert-Butylphenol	ND	0.74±0.25^{a}	0.68±0.05^{a}	0.34±0.04^{ab}	0.42±0.09^{ab}	0.37±0.01^{ab}
Nonanal	0.52±0.04^{a}	0.64±0.14^{a}	0.54±0.13^{a}		0.39±0.06^{ab}	0.42±0.04^{ab}
Heptyl Isobutyl Ketone	1.16±0.09^{a}	0.87±0.14^{ab}	0.63±0.10^{bc}	0.39±0.06^{c}	0.37±0.12^{c}	0.52±0.01^{bc}
Decanal	0.44±0.16^{a}	0.29±0.06^{ab}	0.10±0.03^{b}	0.25±0.14^{ab}	0.14±0.04^{b}	0.14±0.01^{b}
2-Hexadecanol	0.26±0.10^{a}	ND	ND	ND	ND	ND
1-Heptatriacotanol	0.05±0.02^{a}	ND	ND	ND	ND	ND
Decyl Formate	0.11±0.03^{a}	ND	ND	ND	ND	ND
2-Tridecanone	0.43±0.14^{a}	0.19±0.02^{ab}	ND	ND	ND	ND
Hexanoic Acid	0.39±0.08^{b}	0.22±0.06^{b}	0.38±0.01^{b}	0.38±0.01^{b}	0.55±0.03^{b}	1.01±0.16^{a}
Butanedioic Acid Ethyl 3-Methylbutyl Ester	0.08±0.01^{ab}	0.09±0.01^{ab}	0.06±0.01^{b}	0.06±0.01^{b}	0.10±0.01^{ab}	0.11±0.01^{a}
1-Tetradecanol	0.21±0.02^{a}	ND	ND	ND	ND	ND
Palmitic Acid Ethyl Ester	0.24±0.05^{a}	0.18±0.09^{a}	0.10±0.02^{a}	0.25±0.02^{a}	0.18±0.02^{a}	0.18±0.02^{a}
2,6-Di-Tert-Butylhydroquinone	0.24±0.01^{ab}	0.19±0.01^{ab}	0.14±0.01^{ab}	ND	ND	0.25±0.02^{a}
Phenylacetaldehyde	ND	0.06±0.01^{a}	ND	0.06±0.01^{a}	ND	ND

Ethyl Trans-4-Decenoate	ND	0.10±0.01[a]	0.07±0.01[ab]	ND	ND	ND
Ethyl Undecanoate	ND	0.05±0.01[a]	ND	ND	ND	ND
Decyl Alcohol	ND	0.10±0.01[a]	ND	ND	ND	ND
2-Undecanone	ND	ND	0.20±0.03[a]	ND	ND	ND
Ethyl Phenylacetate	ND	ND	0.06±0.01[a]	ND	ND	ND
Methyl-2-Butyl-Caproate	ND	ND	ND	0.14±0.04[a]	ND	ND
2-Benzylpropionic Acid	ND	ND	ND	0.05±0.01[b]	0.08±0.01[ab]	0.10±0.01[a]
Decanoic Acid	ND	ND	0.33±0.10[a]	0.14±0.01[ab]	0.24±0.01[ab]	ND
4-Hydroxybutyric Acid	ND	ND	ND	ND	0.47±0.08[a]	0.49±0.12[a]

Note: [a], [b], and [c] indicate significance analysis.
